# Mental health outcomes of encephalitis: An international web‐based study

**DOI:** 10.1111/ene.16083

**Published:** 2023-10-05

**Authors:** Matt Butler, Yasmin Abdat, Michael Zandi, Benedict D. Michael, Ester Coutinho, Timothy R. Nicholson, Ava Easton, Thomas A. Pollak

**Affiliations:** ^1^ Neuropsychiatry Research and Education Group King's College London London UK; ^2^ Department of Neuroinflammation University College London Queen Square Institute of Neurology London UK; ^3^ Department of Clinical Infection, Microbiology, and Immunology University of Liverpool Liverpool UK; ^4^ Center for Neuroscience and Cell Biology University of Coimbra Coimbra Portugal; ^5^ Encephalitis Society Malton UK

**Keywords:** autoimmune encephalitis, hypersensitivities, infective encephalitis, mental health, psychiatric

## Abstract

**Background and purpose:**

Acute encephalitis is associated with psychiatric symptoms. Despite this, the extent of mental health problems following encephalitis has not been systematically reported.

**Methods:**

We recruited adults who had been diagnosed with encephalitis of any aetiology to complete a web‐based questionnaire.

**Results:**

In total, 445 respondents from 31 countries (55.1% UK, 23.1% USA) responded. Infectious encephalitis constituted 65.4% of cases, autoimmune 29.7%. Mean age was 50.1 years, 65.8% were female, and median time since encephalitis diagnosis was 7 years. The most common self‐reported psychiatric symptoms were anxiety (75.2%), sleep problems (64.4%), mood problems (62.2%), and unexpected crying (35.2%). Self‐reported psychiatric diagnoses were common: anxiety (44.0%), depression (38.6%), panic disorder (15.7%), and posttraumatic stress disorder (PTSD; 21.3%). Severe mental illnesses such as psychosis (3.3%) and bipolar affective disorder (3.1%) were reported. Self‐reported diagnosis rates were broadly consistent with results from the Psychiatric Diagnostic Screening Questionnaire. Many respondents also reported they had symptoms of anxiety (37.5%), depression (28.1%), PTSD (26.8%), or panic disorder (20.9%) that had not been diagnosed. Rates of psychiatric symptoms did not differ between autoimmune and infectious encephalitis. In total, 37.5% respondents had thought about suicide, and 4.4% had attempted suicide, since their encephalitis diagnosis. More than half of respondents (53.5%) reported they had no, or substandard, access to appropriate mental health care. High rates of sensory hypersensitivities (>75%) suggest a previously unreported association.

**Conclusions:**

This large international survey indicates that psychiatric symptoms following encephalitis are common and that mental health care provision may be inadequate. We highlight a need for proactive psychiatric input.

## INTRODUCTION

Encephalitis is a devastating disorder with a global incidence of 0.5–16/100,000 per year [1]. It is defined by the presence of inflammation in the brain parenchyma associated with clinical evidence of neurological dysfunction [2]. Aetiologies are grouped into two categories: infectious and immune‐mediated [3]. In as many as 37%, the cause is unknown, although improving diagnostics may be reducing this figure [4].

The clinical presentations of acute encephalitis are dependent on factors such as the causative agent and the brain regions affected. Common acute features include headache, seizures, focal neurological signs, movement disorders, personality/behavioural changes, and cognitive impairment/confusion [4].

Although direct comparisons are lacking, it is probable that autoimmune encephalitis is more likely to present with isolated or more prominent psychiatric features in the context of subtle neurological signs. In an early series of N‐methyl‐D‐aspartate (NMDA) receptor (NMDAR) antibody encephalitis, as many as 80% of patients were initially assessed by mental health services [5]. This figure has decreased as awareness of the condition has improved [6], but misdiagnosis encephalitis still occurs in a bidirectional manner [7, 8].

Mortality varies by geographical region but can be up to 30% in infectious encephalitis, and in autoimmune encephalitis it ranges between 12% and 40% [1]. Many more survivors are left with life‐changing neurological injury and dysfunction. Common sequelae include physical, cognitive, emotional, behavioural, and social difficulties [9, 10, 11].

Whereas cognitive sequelae have been explored in some detail, studies have seldom directly addressed mental health outcomes [12, 13]. Of these, the majority have had small sample sizes or focus on a relatively narrow range of symptoms [14, 15, 16, 17, 18, 19], or on specific subtypes of encephalitis in specific population groups (e.g., children) [20] and therefore may be unrepresentative. Further research is thus required to assess mental health outcomes in encephalitis of all aetiologies. To date, there have been no large‐scale data exploring the long‐term mental health outcomes of encephalitis from patients' perspectives.

Furthermore, recent evidence has pointed toward sensory (hyper)sensitivities being associated with acquired brain injury, leading to the suggestion that these may also be a feature in patients who have had encephalitis [21].

In this study, we aimed to characterize self‐reported mental health outcomes in the encephalitis population. We aimed to collect data on self‐reported symptoms and diagnoses, in addition to using a validated diagnostic screening tool for common mental health disorders. We also wished to understand patients' experiences of their initial diagnosis, the management of their postencephalitis symptoms, presence or absence of sensory hypersensitivities, and perceptions about the impact of their encephalitis diagnosis. To achieve this, we created a web‐based questionnaire that was distributed to an international sample of encephalitis patients.

## METHODS

### Design and materials

This was a cross‐sectional observational study codesigned with the Encephalitis Society. A web‐based survey (see Supplementary Information) was created using the platform Qualtrics. Respondents were recruited over 19 weeks through an open access link via social media platforms and mailing lists of the Encephalitis Society. Questions were written in clear English, and medical terminology was avoided where possible.

Respondents were individuals who self‐reported a diagnosis of encephalitis, of any subtype, by a medical professional. Respondents were asked to read the electronic participant information sheet, which detailed the aims and scope of the study. Following this, they were asked to provide consent. A single £50 voucher prize draw was offered to respondents who agreed to provide their email address.

Question themes included basic demographics, encephalitis diagnosis and characteristics, experience with and management of postencephalitis symptoms/diagnoses, and illness perceptions. Questions were in the format of tick‐boxes, visual analogue scales (VAS), and free‐text fields. Where respondents were asked to rate the degree to which they agree to a statement, this was on a scale from 0 representing "strongly disagree" to 50 representing "neither agree nor disagree" to 100 representing "strongly agree".

Participants were asked whether they had experienced any sensory hypersensitivities since their encephalitis diagnosis, and were prompted to answer by sensory modality (lights, sounds, touch, taste, temperature, smell, other).

Questions on mood were adapted from the Maudsley three‐item VAS (M3VAS), which has been validated against the Quick Inventory of Depressive Symptomatology 16‐item scale [22]. Respondents were, for example, asked to rate their current mood over the past 2 weeks on a visual scale of 1–100, where 0 represented "not at all depressed" and 100 represented "extremely depressed".

Respondents were asked questions from the Brief Illness Perception Questionnaire (BIPQ), a validated scale used to assess the cognitive and emotional representations of illness [23]. In this study, questions were modified to change the word "illness" to "postencephalitis symptoms", and respondents were asked to rank each answer on a VAS ranging from, for example, 0 = "no control at all" to 100 = "full control".

Respondents completed the Psychiatric Diagnostic Screening Questionnaire (PDSQ), a brief, self‐report scale designed to screen for 13 of the most common mental health disorders as per the Diagnostic and Statistical Manual of Mental Disorders, 4th edition [24]. The PDSQ responses were scored as per the PDSQ scoring instructions.

### Data analysis

Data were analysed using IBM SPSS Statistics 28. Age was calculated as year of birth to survey year; similarly, length of symptoms and time since diagnosis were calculated as year of occurrence to survey year (2022). Unless otherwise stated, data are presented as mean (±SD) or median (interquartile range [IQR]). Where respondents were given the option to select more than one answer, mutually incompatible answers were removed from analysis.

Descriptive statistics (i.e., frequencies, proportions/percentages, measures of central tendency and dispersion) were used to summarize the data. Chi‐squared tests, independent *t*‐tests, and analysis of variance tests were used where appropriate.

Data visualizations were created in RStudio (version 2023.06.0‐421).

### Ethics

This study conforms with the World Medical Association Declaration of Helsinki. The study was approved by King's College London Research Ethics Committee (Ref: 17778).

## RESULTS

### Demographics

In total, 445 respondents from 31 countries completed the survey (Table 1); 45 were completed by carers and 32 with carer assistance. All 445 respondents reported that they had been diagnosed with encephalitis by a medical professional: 300 (72.8%) by a neurologist, 49 (11.9%) by an infectious diseases doctor, nine (2.2%) by a paediatrician, two (0.5%) by a psychiatrist, and 52 (12.6%) by another type of doctor.

**TABLE 1:** Demographics of respondents from the study.

Characteristic	*n*	%
Gender^a^	444	—
Female	292	65.8%
Male	149	33.6%
Age	444	—
Mean years (SD)	50.1 (15.6)	—
Range, years	18–85	—
Employment status	445	—
Full‐time	97	21.8%
Part‐time	55	12.4%
Self‐employed	30	6.7%
Unemployed	122	27.4%
Retired	95	21.3%
Student	20	4.5%
Other	26	5.8%
Country of residence	445	—
UK and Northern Ireland	245	55.1%
USA	103	23.1%
Australia	34	7.6%
Canada	12	2.7%
Other^b^	51	11.5%

^a^ Two preferred to self‐describe, one preferred not to say.

^b^ "Other" includes Belgium (1), Brazil (1), Croatia (2), Cyprus (1), Dominican Republic (1), Finland (1), France (1), Germany (3), Greece (1), India (4), Indonesia (3), Ireland (6), Israel (1), Italy (4), Lebanon (1), Lesotho (1), Namibia (1), the Netherlands (1), New Zealand (5), Norway (2), Philippines (1), Portugal (2), Romania (1), Singapore (1), South Africa (1), Spain (2), and Switzerland (2).

The mean age was 50.1 years (SD = 15.6, range = 18–85); 292 (65.8%) were female. Most respondents resided in the UK (*n* = 245, 55.1%) or the USA (*n* = 103, 23.1%,), but responses were received from 29 additional countries (2.2% from low‐ and middle‐income countries). In total, 432 (98.2%) had completed mandatory education. More than half (*n* = 241, 54.3%,) were married; 182 (40.9%) were in some form of employment, with more than a quarter unemployed (*n* = 122, 27.4%).

### Acute encephalitis

**FIGURE 1:**
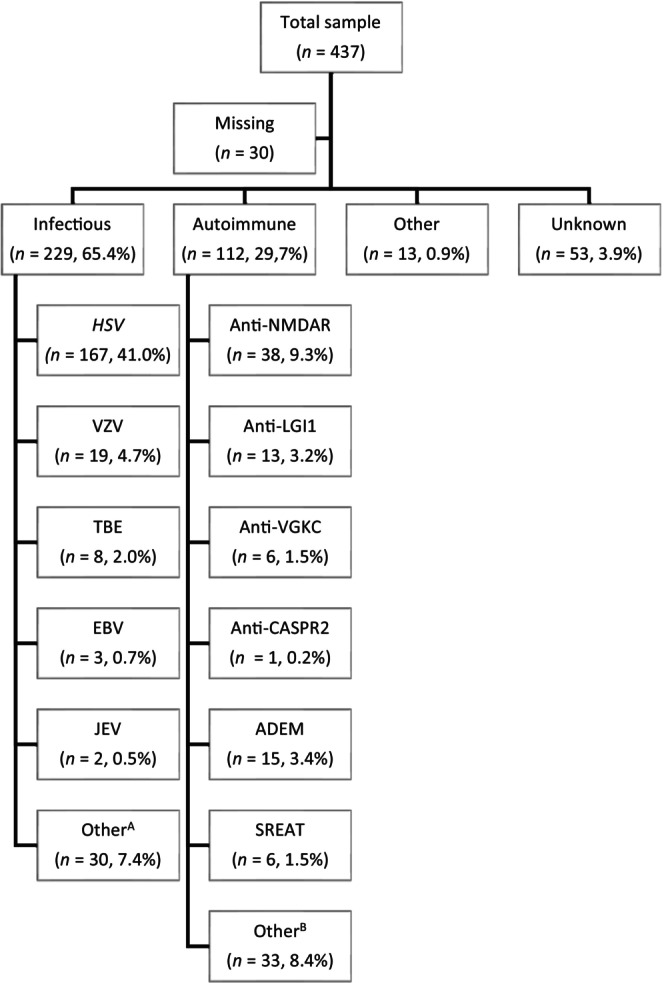
Encephalitis subtypes. Not all respondents submitted responses about the aetiological agent (*n* = 407 included). ^A^Where known, other aetiologies included mumps (*n* = 4), influenza (*n* = 2), COVID‐19 (*n* = 1), equine (*n* = 1), measles (*n* = 1), mycoplasma (*n* = 1), scarlet fever (*n* = 1), and West Nile virus (*n* = 1). ^B^Where known, other aetiologies included Bickerstaff (*n* = 1), acute necrotizing encephalopathy (*n* = 1), anti‐dipeptidyl‐peptidase‐like protein‐6 (*n* = 1), anti‐GAD (*n* = 1), anti‐glial fibrillary acidic protein (*n* = 1), anti‐Hu (*n* = 1), paraneoplastic (*n* = 1), and Rasmussen (*n* = 1). ADEM, acute disseminated encephalomyelitis; CASPR2, contactin‐associated protein‐like 2; EBV, epstein‐barr virus; HSV, herpes simplex virus; JEV, Japanese encephalitis virus; LGI1, leucine‐rich, glioma‐inactivated 1; NMDAR, N‐methyl‐D‐aspartate receptor. SREAT, steroid‐responsive encephalopathy associated with autoimmune thyroiditis; TBE, tick‐borne encephalitis; VGKC, voltage gated potassium channel; VZV, varicella‐zoster virus.

Average time since encephalitis symptoms began was a median of 7.0 years (IQR = 3.0–15.0). Infectious encephalitis constituted 286 (65.4%) cases; the most common causative infectious agent was herpes simplex virus (*n* = 167, 41.0% of all respondents). Autoimmune encephalitis constituted 130 (29.7%), cases with the most common type being anti‐NMDAR encephalitis (*n* = 38, 9.3% of all respondents; Figure 1).

Nearly half (*n* = 209, 47.5%) reported having received an incorrect diagnosis before their final diagnosis. Incorrect diagnoses included physical diagnoses (*n* = 131, 29.8%), psychiatric diagnoses (*n* = 37, 8.4%), both psychiatric and physical diagnoses (*n* = 15, 3.4%), and unknown (*n* = 5, 1.1%). The most common misdiagnoses (*n* = 200) included upper respiratory tract infection (*n* = 24, 12.0%), meningitis (*n* = 17, 8.5%), migraine (*n* = 13, 6.5%), mixed psychiatric diagnoses (*n* = 12, 6.0%), mixed medical diagnoses (*n* = 11, 6.1%), and psychiatric diagnoses not otherwise specified (*n* = 11, 6.1%).

#### Severity of acute illness

Of valid responses (*n* = 442), 268 (60.6%) reported being admitted to a general hospital ward when their encephalitis first started and 40 (9.0%) to intensive care (ITU) or high‐dependency care, and 53 (12.0%) reported they were not admitted to hospital. Thirty respondents (6.8%) were admitted to a psychiatric hospital. Overall, there was no effect of severity based on admission type on likelihood of developing a psychiatric outcome (*z*‐score < 2.0 on chi‐squared tests for all mental health diagnoses for patients admitted to ITU).

### Psychiatric symptoms and diagnoses

**FIGURE 2:**
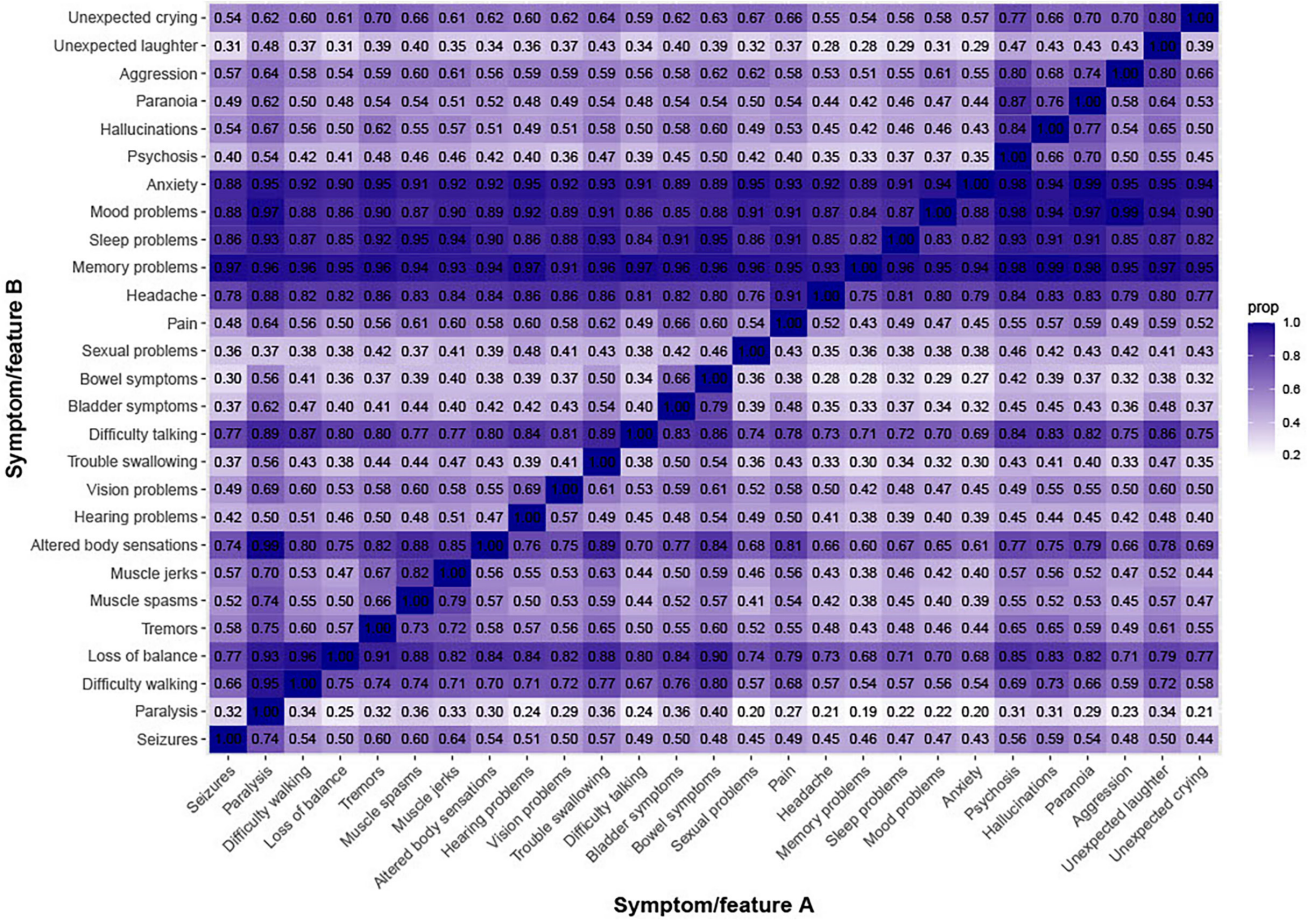
Conditional probability matrix of symptom co‐occurrence. Numbers in the grid indicate the proportion (prop) of respondents with symptom/feature A who also had symptom/feature B. The strength of the proportion is represented by the colour of the square between symptoms; darker colours indicate stronger co‐occurrence of a symptom/feature A with a symptom/feature B. Symptoms were labeled as present if they had occurred at any time since the encephalitis diagnosis.

In total, 402 (90.4%) indicated that they had at least one current psychiatric symptom. Symptoms, both physical and mental, were commonly co‐occurring (Figure 2). Self‐reported psychiatric diagnoses made by a medical professional are presented in Table 2. Numbers of respondents that felt they were suffering from a psychiatric diagnosis that had not been formally diagnosed are reported in Supplementary Tables S1–S3.

**TABLE 2:** Formal mental health or psychiatric diagnoses in respondents.

Diagnosis	*n*	Yes, currently	Yes, in the past, after my encephalitis diagnosis	Yes, in the past, before my encephalitis diagnosis	No
Depression	409	101 (24.7%)	57 (13.9%)	34 (8.3%)	217 (53.1%)
Anxiety	402	127 (31.6%)	50 (12.4%)	35 (8.7%)	190 (47.3%)
Panic disorder	396	32 (8.1%)	30 (7.6%)	15 (3.8%)	319 (80.6%)
Posttraumatic stress disorder	396	43 (10.9%)	41 (10.4%)	14 (3.5%)	298 (75.3%)
Obsessive–compulsive disorder	397	25 (6.3%)	15 (3.8%)	7 (1.8%)	350 (88.2%)
Psychotic disorders	400	7 (1.8%)	6 (1.5%)	9 (2.3%)	378 (94.5%)
Bipolar disorder	398	9 (2.3%)	3 (0.8%)	6 (1.5%)	380 (95.4%)
Personality disorder	398	18 (4.5%)	13 (3.3%)	10 (2.5%)	357 (89.7%)
Impulse control disorder	397	8 (2.0%)	13 (3.3%)	1 (0.3%)	375 (94.5%)
Alcohol dependence	398	7 (1.8%)	7 (1.8%)	5 (1.3%)	379 (95.2%)
Drug dependence	397	7 (1.8%)	2 (0.5%)	4 (1.0%)	384 (96.7%)
Other	301	3 (1.0%)	3 (1.0%)	4 (1.3%)	291 (96.7%)

When asked to rate the degree to which they felt their encephalitis was the cause of their mental health problems (0 = not at all, 100 = entirely), median response was 80.0 (IQR = 50.0–100.0).

In total, 98 of 445 (22.0%) of respondents were currently under a community mental health team (secondary care), and 215 of 445 (48.3%) had undergone some form of talking therapy.

Of those who responded (*n* = 413), 24.2% felt they had not had access to appropriate mental health care, and a further 29.3% thought the access to mental health care could have been better; 28.8% felt they had access to appropriate mental health care, and the remaining 17.7% had not looked; patients in the UK fared the worst (for breakdown by country please see Supplementary Information).

In total, 37.5% of respondents had thought about suicide, and 4.4% of all respondents had attempted suicide since their encephalitis diagnosis.

#### Psychiatric diagnostic screening questionnaire

Using the cutoff scores provided in the PDSQ manual, participants met screening criteria in the following frequencies: social phobia, 161 of 347 (46.4%); depression, 143 of 347 (41.2%); obsessive–compulsive disorder (OCD), 136 of 346 (39.3%); posttraumatic stress disorder (PTSD), 118 of 347 (34.0%); generalized anxiety disorder (GAD), 111 of 347 (32.0%); agoraphobia, 103 of 347 (29.7%); psychosis, 88 of 347 (25.4%); panic disorder, 84 of 347 (24.2%); alcohol use disorder, 57 of 347 (16.4%); eating disorder, 36 of 346 (10.4%); and drug use disorder, 12 of 347 (3.5%).

Concordance of PDSQ scores versus self‐reported diagnoses (formal diagnoses + self‐reported missed diagnoses) was variable: depression, 41.2% versus 44.4%; OCD, 39.3% versus 14.9%; PTSD, 34.0% versus 30.8%; GAD, 24.2% versus 62.4%; psychotic disorder, 25.4% versus 3.9%; panic, 24.2% versus 21.5%; alcohol use disorder, 16.4% versus 4.1%; substance use disorder, 3.5% versus 3.6%. Overall, the PDSQ led to a higher rate of likely diagnoses than did self‐report for OCD, psychotic disorder, and alcohol use disorder.

#### Infectious versus autoimmune

Overall, rates of major self‐reported psychiatric diagnoses and symptoms did not significantly differ between autoimmune and infectious encephalitis on chi‐squared testing (Table 3).

**TABLE 3:** Comparison of psychiatric symptoms and diagnoses split by autoimmune and infectious subtypes.

		Autoimmune			Infectious		
		Yes	Past	No	Yes	Past	No
Symptoms	Anxiety	91 (72.8%)	16 (12.8%)	18 (14.4%)	209 (76.0%)	34 (12.4%)	32 (11.6%)
	Mood problems	66 (52.4%)	36 (28.6%)	24 (19.0%)	181 (66.6%)	47 (17.3%)	44 (16.2%)
	Psychosis	9 (7.5%)	39 (32.5%)	72 (60.0%)	25 (9.6%)	46 (17.8%)	188 (72.6%)
	Aggression	30 (24.8%)	35 (28.9%)	56 (46.3%)	86 (32.2%)	44 (16.5%)	137 (51.3%)
Diagnoses	Anxiety	52 (42.6%)	10 (8.2%)	60 (49.2%)	111 (43.7%)	23 (9.1%)	120 (47.2%)
	Depression	38 (31.4%)	12 (9.9%)	71 (58.7%)	108 (40.5%)	20 (7.6%)	136 (51.9%)
	Panic	11 (9.2%)	5 (4.2%)	104 (86.7%)	43 (17.2%)	8 (3.2%)	199 (79.6%)
	PTSD	21 (17.5%)	4 (3.3%)	95 (79.2%)	53 (21.2%)	10 (4.0%)	188 (74.9%)
	Psychosis	6 (5.0%)	6 (5.0%)	108 (90.0%)	7 (2.8%)	3 (1.2%)	245 (96.1%)
	Bipolar	7 (5.8%)	5 (4.2%)	107 (89.9%)	4 (1.6%)	0 (0.0%)	250 (98.4%)
	Pers. dis.	10 (8.4%)	5 (4.2%)	104 (87.4%)	18 (7.1%)	4 (1.6%)	232 (91.3%)
PDSQ Caseness	Depression	43 (39.8%)			91 (41.6%)		
	PTSD	36 (33.3%)			74 (33.8%)		
	Eating disorder	14 (13.1%)			20 (9.1%)		
	OCD	39 (36.4%)			87 (39.7%)		
	Panic	27 (25.2%)			53 (24.1%)		
	Psychosis	26 (24.3%)			56 (25.5%)		
	Agoraphobia	27 (25.2%)			69 (31.4%)		
	Social phobia	45 (42.1%)			104 (47.3%)		
	Alcohol abuse	14 (13.1%)			39 (17.7%)		
	Drug abuse	4 (3.7%)			6 (2.7%)		
	GAD	25 (23.4%)			82 (37.2%)		

Abbreviations: GAD, generalized anxiety disorder; OCD, obsessive–compulsive disorder; PDSQ, Psychiatric Diagnostic Screening Questionnaire; Pers. dis., personality disorder; PTSD, posttraumatic stress disorder.

#### Brief Illness Perception Questionnaire

**FIGURE 3:**
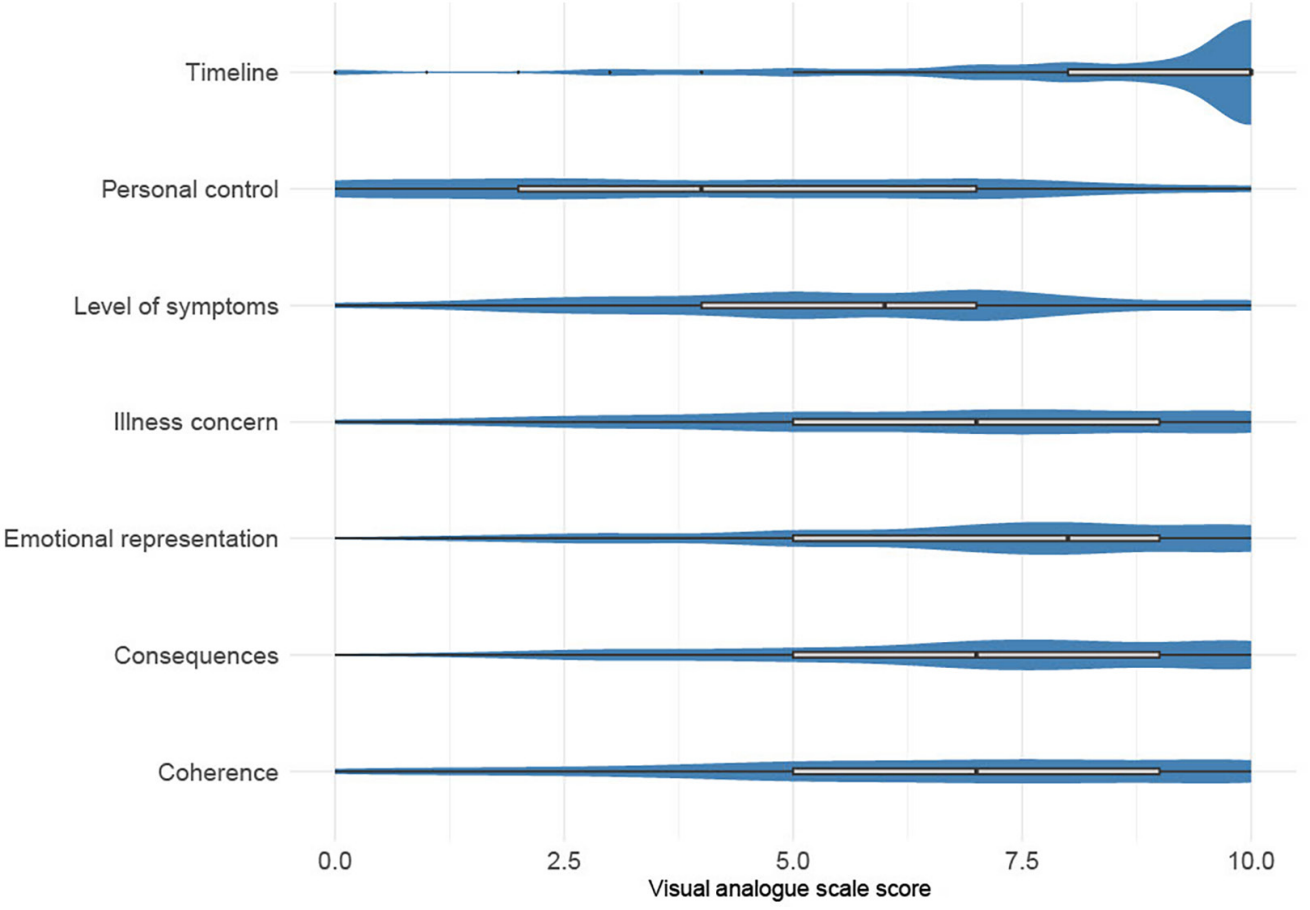
Violin plot of kernel density estimates from the Brief Illness Perception Questionnaire (BIPQ) with overlaid boxplots. There were no differences in mean BIPQ subscores between autoimmune and infectious groups.

Mean and median scores on the BIPQ are summarized in Figure 3.

#### Maudsley VAS

**FIGURE 4:**
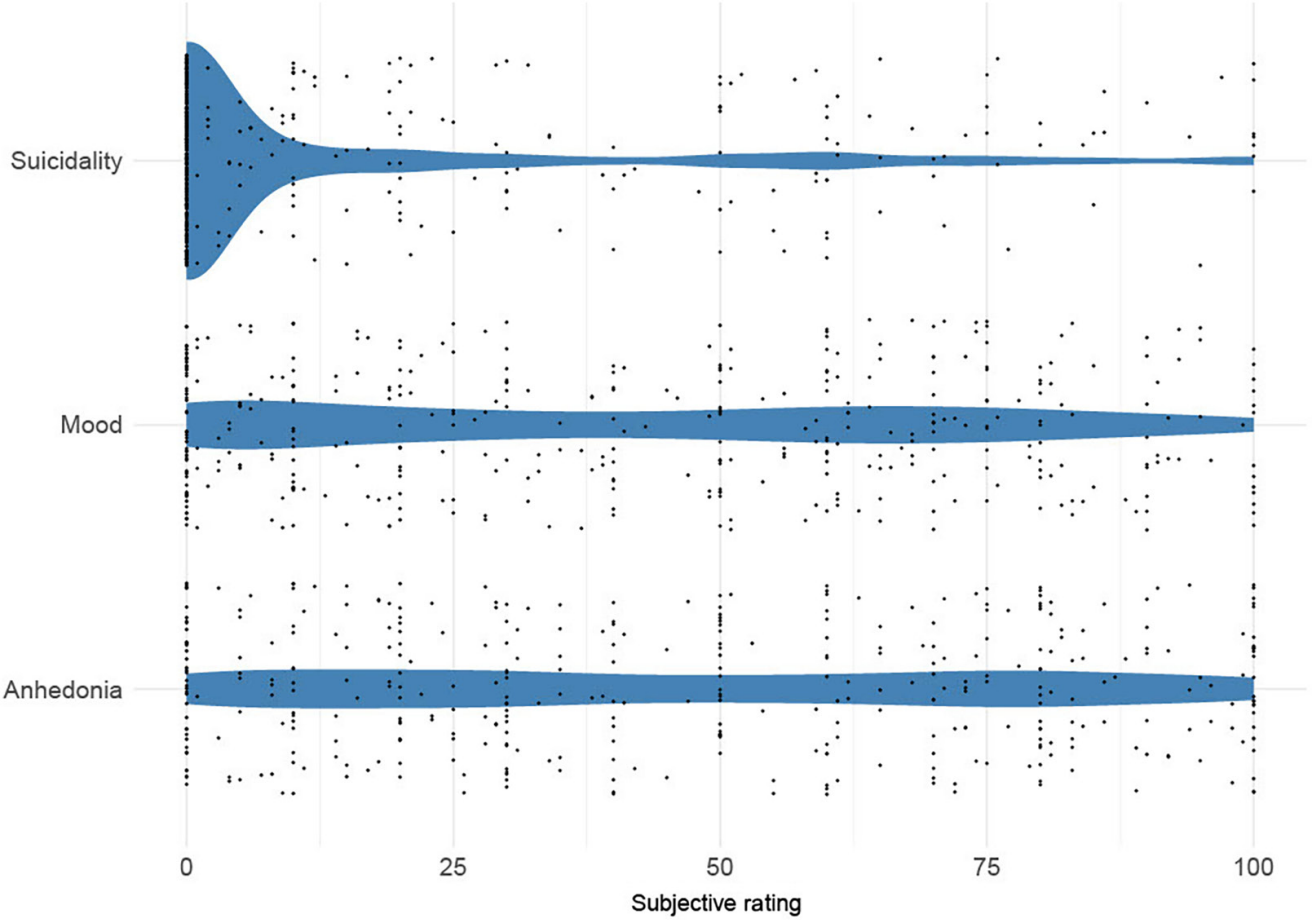
Violin plot of kernel density estimates from the Maudsley three‐item visual analogue scale. Individual participant scores are represented by dots (the random vertical distribution of the dots is a visual aid only). Respondents were asked how severely/frequently they have experienced thoughts or feelings about suicide over the past 2 weeks.

Figure 4 summarizes the results from the M3VAS. Mean (SD) values (of 100) were as follows: suicidality, 15.3 (26.6); mood, 41.8 (32.1); anhedonia, 47.3 (32.5). Median scores (range) were as follows: suicidality, 0.0 (0.0–20.0); mood, 40.0 (10.0–70.0); anhedonia, 50.0 (19.0–77.5). Notably, whereas most respondents experienced relatively low levels of suicidal ideation (modal value = 0, 70.5% of respondents in lowest centile), a minority of patients experienced high levels (7.0% of respondents in highest three centiles).

### Hypersensitivities

In total, 325 of 414 (78.5%) had experienced at least one sensory hypersensitivity; 99 (23.9%) had two hypersensitivities, and 164 (39.6%) three or more hypersensitivities. By modality, there were light hypersensitivities in 233 (56.3%), sound hypersensitivities in 250 (60.4%), touch hypersensitivities in 93 (22.4%), taste hypersensitivities in 95 (22.9%), temperature hypersensitivities in 170 (41.1%), and smell hypersensitivities in 23 (5.6%).

Hypersensitivities were found to have a significant effect on daily life of respondents. On a scale of 0 = "not affecting daily life at all" to 10 = "severely affecting daily life", mean (SD) hypersensitivities were rated as follows: light, 5.3 (2.6); sound, 5.8 (2.6); touch, 5.5 (2.7); taste, 5.1 (2.8); and temperature, 5.8 (2.5). There were no differences between anxiety and depression scores in those with or without hypersensitivities.

### Management of symptoms

The most common treatment used among respondents at any point following encephalitis was medication (64.5% of total sample, *n* = 287); more specifically, nonopiate painkillers (31.5% of total sample, *n* = 140) antidepressants (33.3% of total sample, *n* = 148), benzodiazepines (20.7% of total sample, *n* = 92), sleeping tablets (16.9% of total sample, *n* = 75), opiates (16.2% of total sample, *n* = 72), antipsychotics (11.0% of total sample, *n* = 49), and medicinal cannabis (6.3% of total sample, *n* = 28) had been used. Both answers of "no" and missing responses to these questions were taken as indication that the participant had not used the medication. Self‐reported effectiveness of these treatments is summarized in Table 4.

**TABLE 4:** Self‐reported effectiveness of treatments.

Medication	Unhelpful	Somewhat helpful	Helpful
Antidepressants, *n* = 126	33 (26.2%)	47 (37.3%)	46 (36.5%)
Benzodiazepines, *n* = 81	15 (18.5%)	28 (34.6%)	38 (46.9%)
Antipsychotics, *n* = 44	19 (43.2%)	13 (29.5%)	12 (27.3%)
Sleeping tablets, *n* = 71	16 (22.5%)	33 (46.5%)	22 (31.0%)
Opiates, *n* = 64	12 (18.8%)	28 (43.8%)	24 (37.5%)
Nonopioid painkillers, *n* = 114	25 (21.9%)	40 (35.1%)	49 (43.0%)
Medicinal cannabis, *n* = 77	13 (16.9%)	14 (18.4%)	50 (64.9%)

Other forms of therapy included occupational therapy (*n* = 143, 40.4%), neuropsychological rehabilitation (*n* = 132, 37.3%), cognitive behavioural therapy (*n* = 123, 34.1%), and other psychotherapy (*n* = 107, 30.6%).

Legal nonprescribed substances had also been used to help manage symptoms following encephalitis in 19.1% of respondents and include energy drinks/caffeine (*n* = 27, 7.4%), cannabidiol (CBD; *n* = 27, 7.4%), alcohol (*n* = 19, 5.2%), tobacco (*n* = 10, 2.7%), and e‐cigarettes/nicotine (*n* = 4, 1.1%). Mean (SD) effectiveness of legal substances were as follows (0 = not effective at all, 100 = completely effective): caffeine, 53.6 (32.0); CBD, 67.3 (29.9); alcohol, 42.3 (23.8); tobacco, 49.7 (40.9); and e‐cigarettes/nicotine, 49.7 (47.6).

In total, 6.5% had obtained medication without prescription to treat their symptoms following encephalitis.

In total, 7.5% (*n* = 29) had tried illegal street substances to help manage symptoms following encephalitis, which included cannabis (*n* = 21, 5.4%), psilocybin (*n* = 4, 1.0%), and cocaine (*n* = 3, 0.8%).

## DISCUSSION

This study represents the largest international survey on psychiatric symptoms following encephalitis, acquiring data on an unprecedented breadth of psychiatric symptomatology in a markedly underresearched patient group. Responses indicated high rates of self‐reported mental health symptoms and diagnoses, with many reporting that they had not received adequate psychiatric care following encephalitis. The high rates of psychiatric disorders following encephalitis likely have multiple overlapping causal factors, which include pathogenic brain effects, medication response, residual symptoms, psychological readjustment, and the repercussions of change in functional status and/or resultant physical disability.

The psychiatric community has become increasingly aware of encephalitis since the first descriptions of NMDAR subtype presenting with psychotic symptoms [25]. Autoimmune encephalitis now represents a differential diagnosis for acute, new onset severe psychiatric presentations including psychosis [8, 26]. The current study found that psychiatric symptoms are also very common in the years following encephalitis, indicating that awareness of mental health associations should not be solely restricted to acute presentations.

There are many data indicating that people with other neurological disorders (select examples include epilepsy, Parkinson disease, and migraine) have comorbid psychiatric disorders at rates much higher than population averages [27]. Traumatic brain injury is associated with the development of both neurological and psychiatric sequelae [28]. The relationship is complex and bidirectional; neurological patients with disorders including depression and anxiety report a higher burden of somatic symptoms [29], and recovery from depression in neurological patients is associated with improvement in overall health status, including physical health [30].

Despite this, numerous studies have shown that physicians in general, and neurologists in particular, do not systematically ask about patient's mental health in routine outpatient clinical encounters [29]. In a 2022 survey of 5500 adults with neurological disorders, 61% were not asked about their mental well‐being within the preceding 3 years [31]. As such, considerable mental health difficulties may be missed.

In line with recent recommendations that liaison psychiatry should adopt proactive models of working [32], we suggest that mental health outcomes in encephalitis could be improved either by ongoing involvement of psychiatry or neuropsychiatry in clinical follow‐up. In one study of long‐term psychosocial outcomes in 61 patients with anti‐NMDAR encephalitis, involvement of psychiatry was associated with eightfold increased odds of returning to work or education [17]. Nevertheless, systematic evaluation of the efficacy of mental health interventions in patients with encephalitis‐associated mental illness is urgently required.

Our data regarding suicidality are deeply concerning. Although suicidality and completed suicide have been observed as a feature of anti‐NMDAR encephalitis in one series [33], this represents a relatively understudied area, given the contribution of suicide to avoidable deaths. In an epidemiological study of >7 million Danish citizens, patients who had had encephalitis were at an increased risk of death by suicide (incident rate ratio = 1.7 vs. no neurological diagnosis); this rate is comparable to individuals with epilepsy, Parkinson disease, and head injury, and is higher than stroke and dementia [34]. This is a concern of considerable urgency and mandates further research as well as increased clinical awareness.

The high rates of sensory hypersensitivities seen in our survey have not previously been reported. Sensory processing issues are common in traumatic brain injury [35], cerebral tumours [36], autism spectrum disorders [37], and migraine [38]. The presence of sensory hypersensitivity in these disorders correlates with depression [36], mental distress [35], and a poorer quality of life [39]. In our study, there was no association between anxiety and depression and sensory hypersensitivities, potentially suggesting diverging pathophysiological mechanisms. Further work is required to more fully elucidate the nature, extent, underlying mechanisms, and impacts of sensory hypersensitivities in people who have had encephalitis.

Misdiagnosis was commonly reported by respondents. In this study, a physical health misdiagnosis was more common than a psychiatric misdiagnosis, likely because of the predominance of infectious encephalitis diagnoses among respondents. Increasing the awareness and understanding of encephalitis among health care professionals is therefore likely to be useful in the general hospital/general practice as well as the mental health setting. Misdiagnosis is bidirectional; a recent study indicated that misdiagnosis occurs in approximately one quarter of patients with autoimmune encephalitis, albeit with significant variance between health care settings. The most common conditions incorrectly diagnosed as autoimmune encephalitis were functional neurologic disorder (FND), neurodegenerative disease, and primary psychiatric disease [7]. FND in particular is likely to be a common misdiagnosis in both directions and may be seen as an additional diagnosis in some cases.

### Strengths and limitations

Strengths of this study include the large international dataset, broad focus, and emphasis on patients' own perspectives. The use of an online platform to complete the questionnaire meant that we could reach an international cohort with limited financial or temporal burdens.

The study has several potential limitations. The responses to the questionnaire were self‐reported. We recruited participants through the social media and mailing lists of the Encephalitis Society, which may not be representative of the entire encephalitis population. The data are likely to be subject to response and recall bias, insofar as more severely physically or cognitively challenged people may have been less able to complete the survey, despite our explicit signposting of the possibility of carers completing it on their behalf. Conversely, however, some individuals who have made a full recovery may be less likely to engage with the Encephalitis Society or with encephalitis‐related content online, and therefore potentially less likely to access or complete our questionnaire. We did not collect information on length of time following encephalitis that respondents developed symptoms. We also did not collect any information on cognition or cognitive complaints.

There are attendant limitations to using screening tools such as the PDSQ, which both may overestimate the prevalence of disorders and not capture others. Finally, the questionnaire was only available in English.

## CONCLUSIONS

Results from this large international survey indicate high rates of self‐reported mental health symptoms and diagnoses following encephalitis. Despite the high rates, many respondents reported that mental health care provision following their encephalitis was inadequate. Overall, these results highlight a need for increased provision of proactive psychiatric care and represent a call to action for increased research on mental health outcomes of encephalitis. Given the treatment responsiveness of many mental health symptoms and diagnoses, this is likely to represent a global opportunity for reducing morbidity and mortality in this challenging condition.

## Funding information

M.B. is a Wellcome Trust Doctoral Clinical Research Fellow (227515/Z/23/Z). B.D.M. is supported to conduct COVID‐19 Neuroscience Research by the UKRI/MRC (MR/V03605X/1). B.D.M. is supported for additional neurological inflammation research due to viral infection by grants from the National Institute for Health Research (award CO‐CIN‐01), Medical Research Council (MC_PC_19059), NIHR Health Protection Research Unit in Emerging and Zoonotic Infections at University of Liverpool, MRC/UKRI (MR/V007181/1), Medical Research Council (MR/T028750/1), and Wellcome (ISSF201902/3). B.D.M. is further supported by the Medical Research Foundation (MRF‐CPP‐R2‐2022‐100003).

## CONFLICT OF INTEREST STATEMENT

A.E. is employed by the Encephalitis Society, which helped design and disseminate the survey.

## Supporting information


DATA S1

TABLE S1–S3

## Data Availability

The data that support the findings of this study are available from the corresponding author upon reasonable request.
