# Supplementary material for: Mental health outcomes of encephalitis: An international web‐based study
Source: Eur J Neurol. 2023 Oct 5;31(1):e16083. doi: 10.1111/ene.16083 (PMC11236046; doi:10.1111/ene.16083)
Supplement: Supplementary file 1 — DATA S1 [file ENE-31-e16083-s001.docx]

Q1 **0**      **Consent**
 **0.1**Based on the KCL REC information above, please click "agree" or "disagree" below to provide your consent to participate in this survey:

- Agree (1)
- Disagree (2)

Skip To: End of Survey If 0      Consent 0.1   Based on the KCL REC information above, please click "agree" or "disagree" b... = Disagree

Q109 **0.1**  Is a carer completing this survey on your behalf?

- No (1)
- No, but my carer is assisting me in completing the survey (2)
- Yes, my carer is completing the survey on my behalf (3)

Display This Question:

If 0.1   Is a carer completing this survey on your behalf? != No

Q110 **0.2**  What is the relationship of the carer to you?

- Parent (1)
- Sibling (2)
- Daughter or Son (8)
- Spouse or partner (3)
- Friend (4)
- Nurse (5)
- NHS carer (9)
- Non-NHS carer (6)
- Other (7) __________________________________________________

Display This Question:

If 0.1   Is a carer completing this survey on your behalf? != No

Q111 **0.3**   How long has your carer known you?

▼ Less than 1 year (1) ... 10 years or more (11)

| Page Break |  |
| --- | --- |

Q2 **1**       **Demographics**
 This section will ask some basic demographic questions that will help us understand a little more about you, your history, and your experience with encephalitis.

Q3 **1.1**Please tell us your gender:

- Male (1)
- Female (2)
- Prefer not to say (3)
- Prefer to self-describe (4) __________________________________________________

| 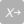 |
| --- |

Q4 **1.2**What is your country of residence?

▼ United Kingdom of Great Britain and Northern Ireland (185) ... Zimbabwe (1357)

Q5 **1.3**What city or town do you live in?

________________________________________________________________

Q6 **1.4**What is the year of your birth?

▼ 2020 (1) ... 1920 (101)

Q7 **1.5**Please tell us about your education:

- Did not complete mandatory education (1)
- Completed mandatory education (2)
- Completed higher education (3)
- Completed tertiary education/university (4)

Q8 **1.6**What is your current relationship status?

- Single (1)
- Married (2)
- Divorced (3)
- Other (4) __________________________________________________

Q9 **1.7**Please tell us about your current employment status:

- Full-time employment (1)
- Part-time employment (2)
- Self-employed (3)
- Unemployed (4)
- Student (5)
- Retired (6)
- Other (7) __________________________________________________

| Page Break |  |
| --- | --- |

Q10 **2**   **Diagnosis and symptoms**
 In this section, we'd like to ask you some specific questions about your condition.

Q11 **2.1**Have you been diagnosed with encephalitis by a medical professional?

- Yes (1)
- No (2)

Q12 **2.1.1**What kind of encephalitis were you diagnosed with?

- Infectious (e.g. viral, bacterial, fungal) (1)
- Autoimmune (2)
- Other (3) __________________________________________________
- Don't know (4)

Q13 **2.1.2**Can you provide more detail about the kind of encephalitis? e.g. the kind of infection (e.g. herpes, VZV, Japanese encephalitis), or the subtype of autoimmune encephalitis (e.g. anti-NMDAR, anti-LGI1).

- Herpes simplex encephalitis (HSV) (4)
- Varicella zoster virus encephalitis (VZV) (5)
- Epstein-Barr virus encephalitis (EBV) (6)
- Japanese encephalitis virus (JEV) (7)
- Tick-borne encephalitis (TBE) (8)
- Anti-NMDAR encephalitis (9)
- Anti-LGI1 encephalitis (10)
- Anti-VGKC encephalitis (11)
- Anti-CASPR2 encephalitis (19)
- Acute disseminated encephalomyelitis (ADEM) (20)
- Steroid-responsive encephalopathy associated with autoimmune thyroiditis (SREAT)/Hashimoto’s encephalopathy’ (21)
- Other infective encephalitis: (12) __________________________________________________
- Other autoimmune encephalitis: (14) __________________________________________________
- Other (18) __________________________________________________
- Unknown (15)
- Don't know (16)

Q14 2.1.3   Who diagnosed your encephalitis?

- Neurologist (1)
- Infectious diseases doctor (2)
- Paediatrician (3)
- Psychiatrist (4)
- Another type of doctor (5)
- Someone else (7) __________________________________________________
- Don't know (6)

Display This Question:

If 2.1.3   Who diagnosed your encephalitis?   = Another type of doctor

Q15 **2.1.4**Which other type of doctor diagnosed your encephalitis?

________________________________________________________________

| 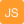 |
| --- |

Q16 **2.2**   What was the approximate month and year that you experienced your first symptoms of encephalitis?

|  | Month | Year |
| --- | --- | --- |
|  |  |  |
| Please Select: (1) | ▼ January (1 ... December (12) | ▼ 1900 (1 ... 2021 (122) |

| 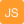 |
| --- |

Q17 **2.3**What was the approximate month and year that you were diagnosed with encephalitis?

|  | Month | Year |
| --- | --- | --- |
|  |  |  |
| Please Select: (1) | ▼ January (1 ... December (12) | ▼ 1900 (1 ... 2021 (122) |

Q18 **2.4**  Do you feel you were given an incorrect diagnosis before you received the correct diagnosis of encephalitis?

- Yes (1)
- No (2)

Display This Question:

If 2.4   Do you feel you were given an incorrect diagnosis before you received the correct diagnosis... = Yes

Q19 2.4.1   What was the incorrect diagnosis that you received?

________________________________________________________________

Q20 **2.5**When your encephalitis first started, did you experience any of the following: 

|  | Yes (1) | No (2) |
| --- | --- | --- |
| Seizures (1) |  |  |
| Paralysis (of limbs or face) (2) |  |  |
| Difficulty walking (3) |  |  |
| Loss of balance (4) |  |  |
| Tremors (5) |  |  |
| Muscle spasms ('dystonia') (6) |  |  |
| Muscle jerks ('myoclonus') (7) |  |  |
| Altered bodily sensations (i.e numbness/tingling) (8) |  |  |
| Hearing problems (9) |  |  |
| Vision problems or blindness (10) |  |  |
| Trouble swallowing or a 'lump in the throat' (11) |  |  |
| Difficulties talking, stuttering, or changes in the speed of your talking (12) |  |  |
| Problems with your memory (13) |  |  |
| Sleep problems (14) |  |  |
| Bladder symptoms (pain or difficulty controlling passing use) (15) |  |  |
| Bowel symptoms (pain or difficulty controlling bowels) (16) |  |  |
| Other pain (17) |  |  |
| Headache (18) |  |  |
| Psychosis (19) |  |  |
| Hallucinations (20) |  |  |
| Paranoia (21) |  |  |
| Mood problems (22) |  |  |
| Aggression/anger management problems or violence (23) |  |  |
| Anxiety (24) |  |  |
| Inappropriate or unexpected laughter (25) |  |  |
| Inappropriate or unexpected crying (26) |  |  |
| Sexual problems (27) |  |  |

Q21 **2.6**   When your encephalitis first started, were you admitted to hospital?

- Yes, on a psychiatric ward (1)
- Yes, on a general ward (2)
- Yes, but I am unsure which ward I was admitted to (4)
- Yes, Other (5) __________________________________________________
- No, I was not admitted to hospital (3)

| 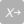 |
| --- |

Q22 2.7   In the months or years following your encephalitis diagnosis, have you experienced any of the following:

|  | Yes currently AND in the past (1) | Yes currently, but not in the past (4) | Yes in the past, but not currently (2) | No (3) |
| --- | --- | --- | --- | --- |
| Seizures (1) |  |  |  |  |
| Paralysis (of limbs or face) (2) |  |  |  |  |
| Difficulty walking (3) |  |  |  |  |
| Loss of balance (4) |  |  |  |  |
| Tremors (5) |  |  |  |  |
| Muscle spasms (‘dystonia’) (6) |  |  |  |  |
| Muscle jerks (‘myoclonus’) (7) |  |  |  |  |
| Altered bodily sensations (i.e. numbness/tingling) (8) |  |  |  |  |
| Hearing problems (9) |  |  |  |  |
| Vision problems or blindness (10) |  |  |  |  |
| Trouble swallowing or a ‘lump in the throat’ (11) |  |  |  |  |
| Difficulties talking, stuttering, or changes in the speed of your talking (12) |  |  |  |  |
| Problems with your memory (13) |  |  |  |  |
| Sleep problems (14) |  |  |  |  |
| Bladder symptoms (pain or difficulty controlling passing use) (15) |  |  |  |  |
| Bowel symptoms (pain or difficulty controlling bowels) (16) |  |  |  |  |
| Other pain (17) |  |  |  |  |
| Headache (18) |  |  |  |  |
| Psychosis (19) |  |  |  |  |
| Hallucinations (20) |  |  |  |  |
| Paranoia (21) |  |  |  |  |
| Mood problems (22) |  |  |  |  |
| Aggression/anger management problems or violence (23) |  |  |  |  |
| Anxiety (24) |  |  |  |  |
| Inappropriate or unexpected laughter (25) |  |  |  |  |
| Inappropriate or unexpected crying (26) |  |  |  |  |
| Sexual problems (27) |  |  |  |  |

Q23 **2.8**Can you think of anything which makes your post-encephalitis symptoms better? (e.g. good sleep, relaxation, exercise)

- Good sleep (4)
- Poor sleep (12)
- Healthy diet (11)
- Unhealthy diet (13)
- Relaxation (5)
- Stress (15)
- Physical exercise (6)
- Physical illness (14)
- Alcohol (7)
- Meditation (8)
- Social interaction (9)
- Other (10) __________________________________________________

Q24 **2.9**Can you think of anything which makes your post-encephalitis symptoms worse? (e.g. poor sleep, physical exercise, stress)

- Good sleep (4)
- Poor sleep (12)
- Healthy diet (13)
- Unhealthy diet (8)
- Relaxation (14)
- Stress (15)
- Physical exercise (5)
- Physical illness (16)
- Alcohol (9)
- Meditation (11)
- Social interaction (10)
- Other (7) __________________________________________________

| Page Break |  |
| --- | --- |

Q103 **2.10**Have you had COVID-19?

- Yes, I have had a positive test (1)
- Yes, I have had symptoms and a doctor diagnosed me (2)
- I have had symptoms but have not been tested or diagnosed by a doctor (3)
- No (4)

Display This Question:

If 2.10   Have you had COVID-19? != No

Q104 **2.10.1**If yes, have you been hospitalised with COVID-19?

- Yes, including Intensive Care Unit (1)
- Yes but not to an Intensive Care Unit (2)
- No (3)

Q106 **2.11**Since the start of the COVID-19 pandemic, have the following symptoms been affected?

|  | Worse (1) | Stayed the same (2) | Better (3) | Not applicable (4) |
| --- | --- | --- | --- | --- |
| Seizures (1) |  |  |  |  |
| Paralysis (of limbs or face) (2) |  |  |  |  |
| Loss of balance (3) |  |  |  |  |
| Tremors (4) |  |  |  |  |
| Muscle spasms (dystonia) (5) |  |  |  |  |
| Muscle jerks ('myoclonus') (7) |  |  |  |  |
| Altered bodily sensations (i.e numbness/tingling) (8) |  |  |  |  |
| Hearing problems (9) |  |  |  |  |
| Vision problems or blindness (10) |  |  |  |  |
| Trouble swallowing or a 'lump in the throat' (11) |  |  |  |  |
| Difficulties talking, stuttering, or changes in the speed of your talking (12) |  |  |  |  |
| Problems with your memory (13) |  |  |  |  |
| Sleep problems (14) |  |  |  |  |
| Bladder symptoms (pain or difficulty controlling passing use) (15) |  |  |  |  |
| Bowel symptoms (pain or difficulty controlling bowels) (16) |  |  |  |  |
| Other pain (17) |  |  |  |  |
| Headache (18) |  |  |  |  |
| Psychosis (19) |  |  |  |  |
| Hallucinations (20) |  |  |  |  |
| Paranoia (21) |  |  |  |  |
| Mood problems (22) |  |  |  |  |
| aggression/anger management problems or violence (23) |  |  |  |  |
| Anxiety (24) |  |  |  |  |
| Inappropriate or unexpected laughter (25) |  |  |  |  |
| Inappropriate or unexpected crying (26) |  |  |  |  |
| Sexual problems (27) |  |  |  |  |

Q107 **2.12**Has the COVID-19 pandemic affected your post-encephalitis symptoms in any other ways?

________________________________________________________________

________________________________________________________________

________________________________________________________________

________________________________________________________________

________________________________________________________________

| Page Break |  |
| --- | --- |

Q25 **3**   **Other medical and mental health conditions** **In this section we will ask you about any other conditions that you may suffer from.**

Q26 **3.1**Do you have any other medical conditions, or things that you see your doctor about, other than encephalitis? (e.g. asthma, diabetes, high blood pressure, headaches, back pain)

________________________________________________________________

Q27  **3.2**Do you have any formal mental health or psychiatric diagnosis (diagnosed by a health professional):

|  | Yes currently (1) | Yes in the past but after my encephalitis diagnosis (2) | Yes in the past but before my encephalitis diagnosis (3) | No (4) |
| --- | --- | --- | --- | --- |
| Depression (1) |  |  |  |  |
| Anxiety (2) |  |  |  |  |
| Panic disorder (3) |  |  |  |  |
| Post traumatic stress disorder (4) |  |  |  |  |
| Obsessive compulsive disorder (5) |  |  |  |  |
| Psychotic disorders (including schizophrenia and schizoaffective disorders) (6) |  |  |  |  |
| Bipolar affective disorder (7) |  |  |  |  |
| Personality disorder (8) |  |  |  |  |
| Impulse control disorder (9) |  |  |  |  |
| Alcohol dependence (10) |  |  |  |  |
| Drug dependence (11) |  |  |  |  |
| Other (12) |  |  |  |  |

Q28 **3.3**Do you feel that you currently suffer from a mental health or psychiatric diagnosis that has NOT been diagnosed by a health professional?

|  | Yes currently (1) | Yes, in the past (4) | No (3) |
| --- | --- | --- | --- |
| Depression (1) |  |  |  |
| Anxiety (2) |  |  |  |
| Panic (3) |  |  |  |
| Post traumatic stress disorder (4) |  |  |  |
| Obsessive compulsive disorder (5) |  |  |  |
| Psychotic disorders (including schizophrenia and schizoaffective disorders) (6) |  |  |  |
| Bipolar affective disorder (7) |  |  |  |
| Personality disorder (8) |  |  |  |
| Impulse control disorder (9) |  |  |  |
| Alcohol dependence (10) |  |  |  |
| Drug dependence (11) |  |  |  |
| Other (12) |  |  |  |

Q29 **3.4**Please move the slider to indicate to what extent do you feel that the mental health problems that you have outlined in the previous two questions were caused by your encephalitis? (0 = not at all caused by encephalitis, 100 = entirely caused by encephalitis)

|  | 0 | 10 | 20 | 30 | 40 | 50 | 60 | 70 | 80 | 90 | 100 |
| --- | --- | --- | --- | --- | --- | --- | --- | --- | --- | --- | --- |

| The extent to which you feel that the mental health problems selected in section 3.2 and 3.3 are caused by your encephalitis () | 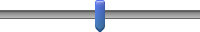 |
| --- | --- |

Q30 **3.5**Are you currently under the care of a psychiatrist or a community mental health team?

- Yes (1)
- No (2)

Q31 **3.6**Since your encephalitis diagnosis, have you seen a psychotherapist or psychologist for talking therapy to address any of the above problems?

- Yes (1)
- No (2)

Q32 **3.7**Do you feel that you have access to appropriate healthcare for your mental health and well-being?

- Yes (1)
- Yes, but it could be better (2)
- No (3)
- I haven't looked (4)

Q33 **3.8   Quality of your mood:** Place the marker on the line to indicate how your mood has been over the past 2 weeks (0 = Not depressed at all, 100 = Extremely depressed)

|  | Not at all depressed | Extremely depressed |
| --- | --- | --- |

|  | 0 | 10 | 20 | 30 | 40 | 50 | 60 | 70 | 80 | 90 | 100 |
| --- | --- | --- | --- | --- | --- | --- | --- | --- | --- | --- | --- |

| Quality of your mood in the past 2 weeks. () | 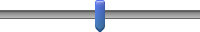 |
| --- | --- |

Q34 **3.9   Experience of pleasure:**
 Place the marker on the line to indicate your ability to enjoy life activities (e.g. work, family and friends, hobbies, television, books or magazines, meals etc.) over the past 2 weeks (0 = Fully able to enjoy any activities, 100 = completely unable to enjoy all activities)

|  | Fully able to enjoy any activities | Completely unable to enjoy all activities |
| --- | --- | --- |

|  | 0 | 10 | 20 | 30 | 40 | 50 | 60 | 70 | 80 | 90 | 100 |
| --- | --- | --- | --- | --- | --- | --- | --- | --- | --- | --- | --- |

| Ability to enjoy life activities that you are still able to do, over the past 2 weeks () | 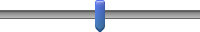 |
| --- | --- |

Q113 **3.10   Experience of suicidal thoughts and feelings:**
Place the marker on the line to indicate how severely/frequently you have experienced thoughts or feelings about suicide over the past 2 weeks (0 = Not at all suicidal, 100 = Extremely suicidal)

|  | Not at all suicidal | Extremely suicidal |
| --- | --- | --- |

|  | 0 | 10 | 20 | 30 | 40 | 50 | 60 | 70 | 80 | 90 | 100 |
| --- | --- | --- | --- | --- | --- | --- | --- | --- | --- | --- | --- |

| Experience of suicidal thoughts and feelings over the past 2 weeks. () | 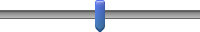 |
| --- | --- |

Q35 **3.11**Have you thought about killing yourself or have you tried to kill yourself since your encephalitis diagnosis?

- Thought about killing myself (1)
- Tried to kill myself (2)
- No (3)

Display This Question:

If 3.11   Have you thought about killing yourself or have you tried to kill yourself since your ence... = Thought about killing myself

Or 3.11   Have you thought about killing yourself or have you tried to kill yourself since your ence... = Tried to kill myself

Or 3.10   Experience of suicidal thoughts and feelings: Place the marker on the line to indicate how... [ Experience of suicidal thoughts and feelings over the past 2 weeks. ] >= 1

Q36 If you live in the UK and need someone to talk to, you can get in touch with the Samaritans about anything that is troubling you, no matter how large or small the issue feels.
Whatever you are going through, call the Samaritans for free at any time, from any phone, on 116 123.
If you live in the UK or internationally, you can contact The Encephalitis Society on +44(0)1653 699599 (9 am to 5 pm (BST), Monday to Thursday, and 9 am to 4.30 pm (BST) on Fridays) or by email: support@encephalitis.info
Additionally, we encourage you to contact your family doctor, who will be able to offer further support.

Q37 **3.12**Since your encephalitis diagnosis, have you suffered from any of the following sensory hypersensitivities?

- Lights (1)
- Sounds (2)
- Touch (5)
- Taste (6)
- Temperature (7)
- Other (3) __________________________________________________
- No (4)

Display This Question:

If 3.12   Since your encephalitis diagnosis, have you suffered from any of the following sensory hyp... = Lights

Q38 **3.12.1**Please indicate how much light hypersensitivity affects your daily life (0 = not affecting daily life at all, 10 = severely affecting daily life) (tap or click to record an answer).

- 0 (0)
- 1 (1)
- 2 (2)
- 3 (3)
- 4 (4)
- 5 (5)
- 6 (6)
- 7 (7)
- 8 (8)
- 9 (9)
- 10 (10)

Display This Question:

If 3.12   Since your encephalitis diagnosis, have you suffered from any of the following sensory hyp... = Sounds

Q39 3.12.2   Please indicate how much sound hypersensitivity affects your daily life (0 = not affecting daily life at all, 10 = severely affecting daily life) (tap or click to record an answer).

- 0 (0)
- 1 (1)
- 2 (2)
- 3 (3)
- 4 (4)
- 5 (5)
- 6 (6)
- 7 (7)
- 8 (8)
- 9 (9)
- 10 (10)

Display This Question:

If 3.12   Since your encephalitis diagnosis, have you suffered from any of the following sensory hyp... = Touch

Q40 3.12.3   Please indicate how much touch hypersensitivity affects your daily life (0 = not affecting daily life at all, 10 = severely affecting daily life) (tap or click to record an answer).

- 0 (0)
- 1 (1)
- 2 (2)
- 3 (3)
- 4 (4)
- 5 (5)
- 6 (6)
- 7 (7)
- 8 (8)
- 9 (9)
- 10 (10)

Display This Question:

If 3.12   Since your encephalitis diagnosis, have you suffered from any of the following sensory hyp... = Taste

Q41 3.12.4   Please indicate how much the taste hypersensitivity affects your daily life (0 = not affecting daily life at all, 10 = severely affecting daily life) (tap or click to record an answer).

- 0 (0)
- 1 (1)
- 2 (2)
- 3 (3)
- 4 (4)
- 5 (5)
- 6 (6)
- 7 (7)
- 8 (8)
- 9 (9)
- 10 (10)

Display This Question:

If 3.12   Since your encephalitis diagnosis, have you suffered from any of the following sensory hyp... = Temperature

Q42 3.12.5   Please indicate how much temperature hypersensitivity affects your daily life (0 = not affecting daily life at all, 10 = severely affecting daily life) (tap or click to record an answer).

- 0 (0)
- 1 (1)
- 2 (2)
- 3 (3)
- 4 (4)
- 5 (5)
- 6 (6)
- 7 (7)
- 8 (8)
- 9 (9)
- 10 (10)

Display This Question:

If 3.12   Since your encephalitis diagnosis, have you suffered from any of the following sensory hyp... = Other

Or Or 3.12&nbsp; &nbsp;Since your encephalitis diagnosis, have you suffered from any of the following sensory hypersensitivities?&nbsp;<o:p></o:p> Text Response Is Not Empty

Q43 3.12.6   Please indicate how much other hypersensitivities affect your daily life (0 = not affecting daily life at all, 10 = severely affecting daily life) (tap or click to record an answer).

|  | 0 | 1 | 2 | 3 | 4 | 5 | 6 | 7 | 8 | 9 | 10 |
| --- | --- | --- | --- | --- | --- | --- | --- | --- | --- | --- | --- |

| Other () | 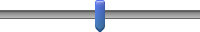 |
| --- | --- |
| Other () | 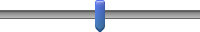 |
| Other () | 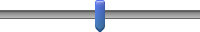 |

Q44 **3.13**Are you reliant on a carer to complete your daily activities (mobilising, washing, eating, getting around etc.)?

- I am totally independent (1)
- I rely on a carer for help with some of my daily activities (2)
- I rely on a carer for help with most of my daily activities (3)
- I am totally dependent on a carer for my daily activities (4)

Display This Question:

If 3.13   Are you reliant on a carer to complete your daily activities (mobilising, washing, eating,... != I am totally independent

Q45
 **3.13.1**Do you think that caring has had an impact on the mental health of your carer? 

- Yes, a big negative impact (1)
- Yes, a slight negative impact (2)
- No impact (3)
- Yes, a slight positive impact (4)
- Yes, a big positive impact (5)

Q46 **3.14**If you wish, please tell us more about the ways in which your post-encephalitis symptoms limit you in your daily life

________________________________________________________________

________________________________________________________________

________________________________________________________________

________________________________________________________________

________________________________________________________________

Q47 3.15   If you wish, please tell us if there is anything about the experience of having/having had encephalitis that has improved or enriched your life

________________________________________________________________

________________________________________________________________

________________________________________________________________

________________________________________________________________

________________________________________________________________

| Page Break |  |
| --- | --- |

Q48 **4**   **Management**
 In this section, we would like to know about your experiences with different types of medications, treatments and self-help strategies for your post-encephalitis symptoms. **Reminder: your answers will remain confidential, please answer to the best of your ability.**

Q49 **4.1**What treatment have you had for your post-encephalitis symptoms?

|  | Yes currently (1) | Yes in the past (2) | No (4) |
| --- | --- | --- | --- |
| Physiotherapy (1) |  |  |  |
| Medication (2) |  |  |  |
| Cognitive behavioural therapy (CBT) (3) |  |  |  |
| Neuropsychological Rehabilitation (8) |  |  |  |
| Other forms of psychotherapy (4) |  |  |  |
| Occupational therapy (5) |  |  |  |
| Other (6) |  |  |  |

Display This Question:

If 4.1   What treatment have you had for your post-encephalitis symptoms?  = Medication [ Yes currently ]

Or 4.1   What treatment have you had for your post-encephalitis symptoms?  = Medication [ Yes in the past ]

Q50 **4.1.1**Which types of medication have you tried? Were they helpful? (If you are responding on your phone, please slide the table to the left to view the whole question)

|  | What types of medication have you tried? | | | Were they helpful? | | | |
| --- | --- | --- | --- | --- | --- | --- | --- |
|  | Yes currently (1) | Yes in the past (2) | No (3) | Helpful (1) | Somewhat helpful (2) | Unhelpful (3) | N/A (4) |
| Antidepressants (e.g. fluoxetine/prozac) (1) |  |  |  |  |  |  |  |
| Benzodiazepines (e.g. diazepam/valium, alprazolam/xanax) (2) |  |  |  |  |  |  |  |
| Antipsychotics (e.g. olanzapine, aripiprazole) (3) |  |  |  |  |  |  |  |
| Sleeping tablets (e.g. zolpidem/ambien) (4) |  |  |  |  |  |  |  |
| Opiates (e.g. co-codamol, codeine, tramadol, morphine) (5) |  |  |  |  |  |  |  |
| Non-opiate painkillers (e.g. ibuprofen) (6) |  |  |  |  |  |  |  |
| Medicinal cannabis/marijuana (in any form) (7) |  |  |  |  |  |  |  |
| Other (8) |  |  |  |  |  |  |  |

Q51 **4.2**Have you ever used legal non-prescription substances to help with your post-encephalitis symptoms?

- Yes (1)
- No (2)
- Don't know (3)

Display This Question:

If 4.2   Have you ever used legal non-prescription substances to help with your post-encephalitis sy... = Yes

Q52 4.2.1   If yes, what have you tried?

- Energy drinks/caffeine (1)
- Alcohol (2)
- Tobacco (including snuff/dip) (3)
- e-cigarettes/nicotine (4)
- CBD (Cannabidiol) (5)
- Solvents (glue) (6)
- Other (7) __________________________________________________

Display This Question:

If 4.2.1   If yes, what have you tried?  = Energy drinks/caffeine

Q53 Please move the slider to indicate how effective you found energy drinks/caffeine in the management of your post-encephalitis symptoms (0 = not at all effective, 100 = extremely effective) (tap or click to record an answer).

|  | 0 | 10 | 20 | 30 | 40 | 50 | 60 | 70 | 80 | 90 | 100 |
| --- | --- | --- | --- | --- | --- | --- | --- | --- | --- | --- | --- |

| Effectiveness of energy drinks/caffeine in the management of your post-encephalitis symptoms () | 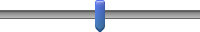 |
| --- | --- |

Display This Question:

If 4.2.1   If yes, what have you tried?  = Alcohol

Q54 Please move the slider to indicate how effective you found alcohol in the management of your post-encephalitis symptoms (0 = not at all effective, 100 = extremely effective) (tap or click to record an answer).

|  | 0 | 10 | 20 | 30 | 40 | 50 | 60 | 70 | 80 | 90 | 100 |
| --- | --- | --- | --- | --- | --- | --- | --- | --- | --- | --- | --- |

| Effectiveness of alcohol in the management of your post-encephalitis symptoms () | 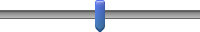 |
| --- | --- |

Display This Question:

If 4.2.1   If yes, what have you tried?  = Tobacco (including snuff/dip)

Q55 Please move the slider to indicate how effective you found tobacco (including snuff/dip) in the management of your post-encephalitis symptoms (0 = not at all effective, 100 = extremely effective) (tap or click to record an answer).

|  | 0 | 10 | 20 | 30 | 40 | 50 | 60 | 70 | 80 | 90 | 100 |
| --- | --- | --- | --- | --- | --- | --- | --- | --- | --- | --- | --- |

| Effectiveness of tobacco (including snuff/dip) in the management of your post-encephalitis symptoms () | 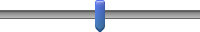 |
| --- | --- |

Display This Question:

If 4.2.1   If yes, what have you tried?  = e-cigarettes/nicotine

Q56 Please move the slider to indicate how effective you found e-cigarettes/nicotine in the management of your post-encephalitis symptoms (0 = not at all effective, 100 = extremely effective) (tap or click to record an answer).

|  | 0 | 10 | 20 | 30 | 40 | 50 | 60 | 70 | 80 | 90 | 100 |
| --- | --- | --- | --- | --- | --- | --- | --- | --- | --- | --- | --- |

| Effectiveness of e-cigarettes/nicotine in the management of your post-encephalitis symptoms () | 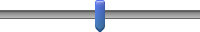 |
| --- | --- |

Display This Question:

If 4.2.1   If yes, what have you tried?  = CBD (Cannabidiol)

Q57 Please move the slider to indicate how effective you found CBD (Cannabidiol) in the management of your post-encephalitis symptoms (0 = not at all effective, 100 = extremely effective) (tap or click to record an answer).

|  | 0 | 10 | 20 | 30 | 40 | 50 | 60 | 70 | 80 | 90 | 100 |
| --- | --- | --- | --- | --- | --- | --- | --- | --- | --- | --- | --- |

| Effectiveness of CBD (cannabidiol) in the management of your post-encephalitis symptoms () | 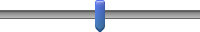 |
| --- | --- |

Display This Question:

If 4.2.1   If yes, what have you tried?  = Solvents (glue)

Q58 Please move the slider to indicate how effective you found solvents (glue) in the management of your post-encephalitis symptoms (0 = not at all effective, 100 = extremely effective) (tap or click to record an answer).

|  | 0 | 10 | 20 | 30 | 40 | 50 | 60 | 70 | 80 | 90 | 100 |
| --- | --- | --- | --- | --- | --- | --- | --- | --- | --- | --- | --- |

| Effectiveness of solvents (glue) in the management of your post-encephalitis symptoms () | 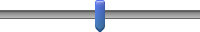 |
| --- | --- |

Display This Question:

If 4.2.1   If yes, what have you tried?  = Other

And And 4.2.1&nbsp; &nbsp;If yes, what have you tried?&nbsp;<o:p></o:p> Other Is Displayed

Q59 Please move the slider to indicate how effective you found any other legal substances stated in section 4.2.1, in the management of your post-encephalitis symptoms (0 = not at all effective, 100 = extremely effective) (tap or click to record an answer).

|  | 0 | 10 | 20 | 30 | 40 | 50 | 60 | 70 | 80 | 90 | 100 |
| --- | --- | --- | --- | --- | --- | --- | --- | --- | --- | --- | --- |

| Substance: () | 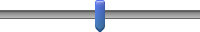 |
| --- | --- |
| Substance: () | 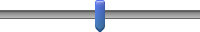 |
| Substance: () | 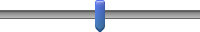 |

Q60 **4.3**Have you ever used prescription medication obtained without a prescription from a doctor (e.g. obtained over the internet) to manage your post-encephalitis symptoms? (reminder: your answers will remain confidential, please answer to the best of your ability).

- Yes (1)
- No (2)
- Don't know (3)

Q61 **4.4**Have you ever undergone any surgical operations or invasive procedures to help with your post-encephalitis symptoms?

- Yes (1)
- No (2)
- Don't know (3)

Display This Question:

If 4.4   Have you ever undergone any surgical operations or invasive procedures to help with your po... = Yes

Q62 4.4.1   If yes, what was this?

________________________________________________________________

63 **4.5**Have you ever used any illegal (street) drugs or unregulated substances to help manage your post-encephalitis symptoms? (reminder: your answers will remain confidential, please answer to the best of your ability)

- Yes (1)
- No (2)
- Don't know (3)

Display This Question:

If 4.5   Have you ever used any illegal (street) drugs or unregulated substances to help manage your... = Yes

Q64 4.5.1   If yes, what illegal (street) substances have you tried?

________________________________________________________________

Display This Question:

If 4.5   Have you ever used any illegal (street) drugs or unregulated substances to help manage your... = Yes

Q65 If you live in the UK and need some friendly and confidential advice about substance misuse, there are many helplines available. 
Please Call **FRANK** for advice regarding drug misuse, on **0300 123 6600**  (available 24 hours a day, 7 days a week) 
or **Drinkline**, for alcohol misuse, on **0300 123 1110** (weekdays 9 am – 8 pm, weekends 11 am – 4 pm).
 
If you live in UK or internationally, you can contact **The Encephalitis Society** on **+44(0)1653 699599** (9 am to 5 pm (BST), Monday to Thursday, and 9 am to 4.30 pm (BST) on Fridays) or by email at: **support@encephalitis.info**.
 
Additionally, we encourage you to contact your family doctor, who will be able to offer further support.

| Page Break |  |
| --- | --- |

Q66 **5**   **Illness model** **In this section we will ask you about what you think about the causes of your encephalitis symptoms.**   Some of the following questions are taken from a commonly used scale that measures ‘illness perceptions’ – the Brief Illness Perception Scale (the ‘BIPQ’) - and is used for physical, functional and psychological disorders. Please note that this is designed for use in disorders with a range of severity, such as kidney disease, so responses to certain questions such as ‘effect on life’ and ‘concern about symptoms’ are expected to vary considerably.

Q67 **5.1**What do you think was the cause of your encephalitis?

________________________________________________________________

Q68 **5.2**Please indicate how much your post-encephalitis symptoms affect your life (tap or click to record an answer)

- 0 (0)
- 1 (1)
- 2 (2)
- 3 (3)
- 4 (4)
- 5 (5)
- 6 (6)
- 7 (7)
- 8 (8)
- 9 (9)
- 10 (10)

Q69 **5.3**Please indicate how long you think your post-encephalitis symptoms will continue for (tap or click to record an answer).

- 0 (0)
- 1 (1)
- 2 (2)
- 3 (3)
- 4 (4)
- 5 (5)
- 6 (6)
- 7 (7)
- 8 (8)
- 9 (9)
- 10 (10)

Q70 **5.4**Please indicate how much control you feel you have over your post-encephalitis symptoms (tap or click to record an answer).

- 0 (0)
- 1 (1)
- 2 (2)
- 3 (3)
- 4 (4)
- 5 (5)
- 6 (6)
- 7 (7)
- 8 (8)
- 9 (9)
- 10 (10)

Q71 5.5   Please indicate the level of post-encephalitis symptoms that you feel that you currently have (tap or click to record an answer).

- 0 (0)
- 1 (1)
- 2 (2)
- 3 (3)
- 4 (4)
- 5 (5)
- 6 (6)
- 7 (7)
- 8 (8)
- 9 (9)
- 10 (10)

Q72 **5.6**Please indicate how concerned you are about your post-encephalitis symptoms (tap or click to record an answer).

- 0 (0)
- 1 (1)
- 2 (2)
- 3 (3)
- 4 (4)
- 5 (5)
- 6 (6)
- 7 (7)
- 8 (8)
- 9 (9)
- 10 (10)

Q73 **5.7**Please indicate how well you feel you understand your post-encephalitis symptoms (tap or click to record an answer).

- 0 (0)
- 1 (1)
- 2 (2)
- 3 (3)
- 4 (4)
- 5 (5)
- 6 (6)
- 7 (7)
- 8 (8)
- 9 (9)
- 10 (10)

Q74 **5.8**Please indicate how much your post-encephalitis symptoms affect you emotionally (e.g. do they make you angry, scared, upset or depressed) (tap or click to record an answer).

- 0 (0)
- 1 (1)
- 2 (2)
- 3 (3)
- 4 (4)
- 5 (5)
- 6 (6)
- 7 (7)
- 8 (8)
- 9 (9)
- 10 (10)

End of Block: Patient information sheet, Informed consent, Questions

Start of Block: PDSQ

Q75 **Section 2**
In this next section, you will be asked about your emotions, moods, thoughts, and behaviours. For each question, click YES in the column next to that question, if it describes how you have been acting, feeling, or thinking. If the item does not apply to you, click NO in the column next to that question.
**PLEASE ANSWER EVERY QUESTION**

Q76 During the past 2 weeks...

|  | During the past 2 weeks... | |
| --- | --- | --- |
|  | Yes (1) | No (2) |
| …did you feel sad or depressed? (1) |  |  |
| …did you feel sad or depressed for most of the day, nearly every day? (2) |  |  |
| …did you get less joy or pleasure from almost all of the things you normally enjoy? (3) |  |  |
| …were you less interested in almost all of the activities you are usually interested in? (4) |  |  |
| …was your appetite significantly smaller then usual nearly every day? (5) |  |  |
| …was your appetite significantly greater then usual nearly every day? (6) |  |  |
| …did you sleep at least 1 to 2 hours less than usual nearly every day? (7) |  |  |
| …did you sleep at least 1 to 2 hours more than usual nearly every day? (8) |  |  |
| …did you feel very jumpy and physically restless, and have a lot of trouble sitting calmly in a chair, nearly every day? (9) |  |  |
| …did you feel tired out nearly every day? (10) |  |  |
| …did you frequently feel guilty about things you have done? (11) |  |  |
| …did you put yourself down and have negative thoughts about yourself nearly every day? (12) |  |  |
| …did you feel like a failure nearly every day? (13) |  |  |
| …did you have problems concentrating nearly every day? (14) |  |  |
| …was decision making more difficult than normal nearly every day? (15) |  |  |
| …did you frequently think of dying in passive ways like going to sleep and not waking up? (16) |  |  |
| …did you wish you were dead? (17) |  |  |
| …did you think you were better off dead? (18) |  |  |
| …did you have thoughts of suicide, even thought you would not really do it? (19) |  |  |
| …did you seriously consider taking your life? (20) |  |  |
| …did you think about a specific way to take your life? (21) |  |  |
| …Have you ever experienced a traumatic event such as combat, rape, assault, sexual abuse, or any other extremely upsetting event? (22) |  |  |
| …Have you ever witnessed a traumatic event such as rape, assault, someone dying in an accident, or any other extremely upsetting incident? (23) |  |  |

Q77 During the past 2 weeks...

|  | During the past 2 weeks... | |
| --- | --- | --- |
|  | Yes (1) | No (2) |
| …did thoughts about a traumatic event frequently pop into your mind? (1) |  |  |
| …did you frequently get upset because you were thinking about a traumatic event? (2) |  |  |
| …were you frequently bothered by memories or dreams of a traumatic event? (3) |  |  |
| …did reminders of a traumatic event cause you to feel intense distress? (4) |  |  |
| …did you try to block out thoughts or feelings related to a traumatic event? (5) |  |  |
| …did you try to avoid activities, places, or people that reminded you of a traumatic event? (6) |  |  |
| …did you have flashbacks, where it felt like you were reliving a traumatic event? (7) |  |  |
| …did reminders of a traumatic event make you shake, break out into a sweat, or have a racing heart? (8) |  |  |
| …did you feel distant and cutoff from other people because of having experienced a traumatic event? (9) |  |  |
| …did you feel emotionally numb because of having experienced a traumatic event? (10) |  |  |
| …did you give up on goals for the future because of having experienced a traumatic event? (11) |  |  |
| …did you keep your guard up because of having experienced a traumatic event? (12) |  |  |
| …were you jumpy and easily startled because of having experienced a traumatic event? (13) |  |  |

Q78 During the past 2 weeks...

|  | During the past 2 weeks... | |
| --- | --- | --- |
|  | Yes (1) | No (2) |
| …did you often go on eating binges (eating a very large amount of food very quickly over a short period of time)? (1) |  |  |
| …did you often feel you could not control how much you were eating during an eating binge? (2) |  |  |
| …did you go on eating binges during which you ate so much that you felt uncomfortably full? (3) |  |  |
| …did you go on eating binges during which you ate a large amount of food even when you didn’t feel hungry? (4) |  |  |
| …did you eat alone during an eating binge because you were embarrassed by how much you were eating? (5) |  |  |
| …did you go on eating binges and then feel disgusted with yourself afterwards? (6) |  |  |
| …were you very upset with yourself because you were going on eating binges? (7) |  |  |
| …to prevent gaining weight from an eating binge did you go on strict diets or exercise excessively? (8) |  |  |
| …to prevent weight gain from an eating binge did you force yourself to vomit or use laxatives or water pills? (9) |  |  |
| …was your weight, or the shape of your body, one of the most important things that affected your opinion of yourself? (10) |  |  |

Q79 During the past 2 weeks...

|  | During the past 2 weeks... | |
| --- | --- | --- |
|  | Yes (1) | No (2) |
| …did you worry obsessively about dirt, germs, or chemicals? (1) |  |  |
| …did you worry obsessively that something bad would happen because you forgot to do something important – like locking the door, turning off the stove, or pulling out the electrical cords of appliances? (2) |  |  |
| …were there things you felt compelled to do over and over (for at least ½ hour per day) that you could not stop doing when you tried? (3) |  |  |
| …were there things you felt compelled to do over and over even though they interfered with getting other things done? (4) |  |  |
| …did you wash and clean yourself or things around you obsessively and excessively? (5) |  |  |
| …did you obsessively and excessively check things or repeat actions over and over again? (6) |  |  |
| …did you count things obsessively and excessively? (7) |  |  |

Q80 During the past 2 weeks...

|  | During the past 2 weeks... | |
| --- | --- | --- |
|  | Yes (1) | No (2) |
| …did you get very scared because your heart was beating fast? (1) |  |  |
| …did you get very scared because you were feeling shaky or faint? (2) |  |  |
| …did you get sudden attacks of intense anxiety or fear that came on from out of the blue, for no reason at all? (3) |  |  |
| …did you get sudden attacks of very intense anxiety or fear during which you thought something terrible might happen, such as your dying, going crazy; or losing control? (4) |  |  |
| …did you have sudden, unexpected attacks of anxiety during which you had three or more of the following symptoms: heart racing or pounding, sweating, shakiness, shortness of breath, nausea, dizziness, or feeling faint? (5) |  |  |
| …did you worry a lot about having unexpected anxiety attacks? (6) |  |  |
| …did you have anxiety attacks that caused you to avoid certain situations or to change your behavior or normal routine (7) |  |  |
| …did you get very scared because you were short of breath? (9) |  |  |

Q81 During the past 2 weeks...

|  | During the past 2 weeks... | |
| --- | --- | --- |
|  | Yes (1) | No (2) |
| …did things happen that you knew were true, but that other people told you were your imagination? (1) |  |  |
| …were you convinced that other people were watching you, talking about you, or spying on you? (2) |  |  |
| …did you think that you were in danger because someone was plotting to hurt you? (3) |  |  |
| …did you think that you had special powers other people didn’t have? (4) |  |  |
| …did you think that some outside force or power was controlling you body or mind? (5) |  |  |
| …did you hear voices that other people didn’t hear, or see things that other people didn’t see? (6) |  |  |

Q82 During the past 2 weeks...

|  | During the past 2 weeks... | |
| --- | --- | --- |
|  | Yes (1) | No (2) |
| …did you regularly avoid any situations because you were afraid they’d cause you to have an anxiety attack? (1) |  |  |
| …did any of the following make you feel fearful, anxious, or nervous because you were afraid you’d have an anxiety attack in the situation? a) going outside far away from home (2) |  |  |
| b) being in crowded places (3) |  |  |
| c) standing in long lines (4) |  |  |
| d) being on a bridge or in a tunnel (5) |  |  |
| e) travelling in a bus, train, or plane (6) |  |  |
| f) driving or riding in a car (7) |  |  |
| g) being home alone (8) |  |  |
| h) being in wide-open spaces (like a park) (9) |  |  |
| …did you almost always get very anxious as soon as you were in any of the above situations? (10) |  |  |
| …did you avoid any of the above situations because they made you feel anxious or fearful? (11) |  |  |

Q83 During the past 2 weeks...

|  | During the past 2 weeks... | |
| --- | --- | --- |
|  | Yes (1) | No (2) |
| …did you worry a lot about embarrassing yourself in front of others? (1) |  |  |
| …did you worry a lot that you might do something to make people think that you were stupid or foolish? (2) |  |  |
| …did you feel very nervous in situations where people might pay attention to you? (3) |  |  |
| …were you extremely nervous in social situations? (4) |  |  |
| …did you regularly avoid any situations because you were afraid you’d do or say something to embarrass yourself? (5) |  |  |
| …did you worry a lot about doing or saying something to embarrass yourself in any of the following situations? a) public speaking (6) |  |  |
| b) eating in front of other people (7) |  |  |
| c) using public restrooms (8) |  |  |
| d) writing in front of others (9) |  |  |
| e) saying something stupid when you were with a group of people (10) |  |  |
| f) asking a question when in a group of people (11) |  |  |
| g) business meetings (12) |  |  |
| h) parties or other social gatherings (13) |  |  |
| …did you almost always get very anxious as soon as you were in any of the above situations? (14) |  |  |
| …did you avoid any of the above situations because they made you feel anxious or fearful? (15) |  |  |

Q84 During the past 2 weeks...

|  | During the past 2 weeks... | |
| --- | --- | --- |
|  | Yes (1) | No (2) |
| …did you think that you were drinking too much? (1) |  |  |
| …did anyone in your family think or say that you were drinking too much, or that you had an alcohol problem? (2) |  |  |
| …did friends, a doctor, or anyone else think or say that you were drinking too much? (3) |  |  |
| …did you think about cutting down or limiting your drinking? (4) |  |  |
| …did you think that you had an alcohol problem? (5) |  |  |
| …because of your drinking did you have problems in your marriage; at your job; with your friends or family; doing household chores; or in any other important area of your life? (6) |  |  |

Q85 During the past 2 weeks...

|  | During the past 2 weeks... | |
| --- | --- | --- |
|  | Yes (1) | No (2) |
| …did you think that you were using drugs too much? (1) |  |  |
| …did anyone in your family think or say that you were using drugs too much, or that you had a drug problem? (2) |  |  |
| …did friends, a doctor, or anyone else think or say that you were using drugs too much? (3) |  |  |
| …did you think about cutting down or limiting your drug use? (4) |  |  |
| …did you think that you had a drug problem? (5) |  |  |
| …because of your drug use did you have problems in your marriage; at your job; with your friends or family; doing household chores; or in any other important area of your life? (6) |  |  |

Q86 During the past 2 weeks...

|  | During the past 2 weeks... | |
| --- | --- | --- |
|  | Yes (1) | No (2) |
| …were you a nervous person on most days? (1) |  |  |
| …did you worry a lot that bad things might happen to you or someone close to you? (2) |  |  |
| …did you worry about things that other people said you shouldn’t worry about? (3) |  |  |
| …were you worried or anxious about a number of things in your daily life on most days? (4) |  |  |
| …did you often feel restless or on edge because you were worrying? (5) |  |  |
| …did you often have problems falling asleep because you were worrying about things? (6) |  |  |
| …did you often feel tension in your muscles because of anxiety or stress? (7) |  |  |
| …did you often have difficulty concentrating because your mind was on your worries? (8) |  |  |
| …were you often snappy or irritable because you were worrying or feeling stressed out? (9) |  |  |
| …was it hard for you to control or stop your worrying on most days (10) |  |  |

Q87 During the past 2 weeks...

|  | During the past 2 weeks... | |
| --- | --- | --- |
|  | Yes (1) | No (2) |
| …have you had a lot of stomach and intestinal problems such as nausea, vomiting, excessive gas, stomach bloating, or diarrhea? (1) |  |  |
| …have you been bothered by aches and pains in many different parts of your body? (2) |  |  |
| Do you get sick more than most people? (3) |  |  |
| Has your physical health been poor most of your life? (4) |  |  |
| Are your doctors usually unable to find a physical cause for your physical symptoms? (5) |  |  |

Q88 During the past 2 weeks...

|  | During the past 2 weeks... | |
| --- | --- | --- |
|  | Yes (1) | No (2) |
| …did you often worry that you might have a serious physical illness? (1) |  |  |
| …was it hard to stop worrying that you have a serious physical illness? (2) |  |  |
| …did your doctor say you didn’t have a serious illness but it was still hard to stop thinking about it? (3) |  |  |
| …did you worry so much about having a serious illness that it interfered with your activities or it caused you problems? (4) |  |  |
| …did you visit the doctor a lot because you were worried that you had a serious physical illness? (5) |  |  |

End of Block: PDSQ

Start of Block: End of survey

Q83 **Follow up**   **Thank you for completing this survey! Your responses will contribute to current research on encephalitis.**   If you have any questions or require more information about this study, please contact us using the following contact details:
 Dr Thomas Pollak at thomas.pollak@kcl.ac.uk,
 or Yasmin Abdat at yasmin.abdat@kcl.ac.uk.   Additionally, if you need further support, please contact The Encephalitis Society at support@encephalitis.info.

 You can access the details of all the organisations mentioned throughout the survey here: [Contact details listed within the questionnaire](https://kcliop.eu.qualtrics.com/CP/File.php?F=F_9WxD09IypRfhPFz)
 If you are happy to, please provide a valid email address for entry into the prize draw and/or to be contacted about future research: [CLICK HERE TO ENTER](https://qualtrics.kcl.ac.uk/jfe/form/SV_43ooxgo3y8x5Kaa)    Any comments or feedback? (not required)

________________________________________________________________

________________________________________________________________

________________________________________________________________

________________________________________________________________

________________________________________________________________

End of Block: End of survey
