# Supplementary material for: Mental health outcomes of encephalitis: An international web‐based study
Source: Eur J Neurol. 2023 Oct 5;31(1):e16083. doi: 10.1111/ene.16083 (PMC11236046; doi:10.1111/ene.16083)
Supplement: Supplementary file 2 — TABLE S1–S3 [file ENE-31-e16083-s002.docx]

| **Level of education** | **440** | **-** |
| --- | --- | --- |
| *Did not complete mandatory education* | *8* | 1.8% |
| *Completed mandatory education* | *104* | 23.6% |
| *Completed higher education* | *109* | 24.8% |
| *Completed tertiary education/university* | *219* | 49.8% |
| **Relationship status** | **444** |  |
| *Single* | *107* | 24.1% |
| *In a relationship* | *27* | 6.1% |
| *Married* | *241* | 54.3% |
| *Divorced/separated* | *42* | 9.5% |
| *Other* | *27* | 6.1% |

*Supplementary table 1: education and relationship status of participants.*

| **Diagnosis** | **N** | **Yes, currently** | **Yes, in the past** | **No** |
| --- | --- | --- | --- | --- |
| Depression | 395 | 78 (19.7%) | 33 (8.4%) | 284 (71.9%) |
| Anxiety | 390 | 120 (30.8%) | 26 (6.7%) | 244 (62.6%) |
| Panic disorder | 388 | 52 (13.4%) | 29 (7.5%) | 307 (79.1%) |
| Post-traumatic stress disorder | 391 | 78 (19.9%) | 27 (6.9%) | 286 (73.1%) |
| Obsessive compulsive disorder | 384 | 33 (8.6%) | 19 (4.9%) | 332 (86.5%) |
| Psychotic disorders | 382 | 8 (2.1%) | 4 (1.0%) | 370 (96.9%) |
| Bipolar disorder | 379 | 7 (1.8%) | 2 (0.5%) | 370 (97.6%) |
| Personality disorder | 383 | 22 (5.7%) | 15 (3.9%) | 346 (90.3%) |
| Impulse control disorder | 385 | 22 (5.7%) | 12 (3.1%) | 351 (91.2%) |
| Alcohol dependence | 386 | 9 (2.3%) | 11 (2.8%) | 366 (94.8%) |
| Drug dependence | 383 | 7 (1.8%) | 5 (1.3%) | 371 (96.9%) |
| Other | 309 | 10 (3.2%) | 3 (1.0%) | 296 (95.8%) |

*Supplementary table 2: Number of respondents that believe they are suffering from a psychiatric diagnosis that has NOT been formally diagnosed by a healthcare professional.*

| Do you feel that you have access to appropriate healthcare for your mental health and well-being? | | | | | | | |
| --- | --- | --- | --- | --- | --- | --- | --- |
|  | | Missing | I haven't looked | No | Yes | Yes, but it could be better | Total |
| Country | Australia | 1 | 4 | 4 | 17 | 8 | 34 |
|  | Belgium | 0 | 0 | 0 | 1 | 0 | 1 |
|  | Brazil | 0 | 0 | 0 | 0 | 1 | 1 |
|  | Canada | 0 | 0 | 5 | 5 | 2 | 12 |
|  | Croatia | 0 | 1 | 0 | 0 | 1 | 2 |
|  | Cyprus | 0 | 0 | 0 | 1 | 0 | 1 |
|  | Dominican Republic | 0 | 0 | 0 | 1 | 0 | 1 |
|  | Finland | 0 | 0 | 0 | 1 | 0 | 1 |
|  | France | 0 | 0 | 0 | 1 | 0 | 1 |
|  | Germany | 0 | 1 | 1 | 1 | 0 | 3 |
|  | Greece | 0 | 0 | 1 | 0 | 0 | 1 |
|  | India | 0 | 0 | 2 | 2 | 0 | 4 |
|  | Indonesia | 0 | 3 | 0 | 0 | 0 | 3 |
|  | Ireland | 0 | 0 | 1 | 2 | 3 | 6 |
|  | Israel | 0 | 0 | 0 | 0 | 1 | 1 |
|  | Italy | 0 | 1 | 1 | 1 | 1 | 4 |
|  | Lebanon | 0 | 1 | 0 | 0 | 0 | 1 |
|  | Lesotho | 0 | 0 | 1 | 0 | 0 | 1 |
|  | Namibia | 0 | 0 | 1 | 0 | 0 | 1 |
|  | Netherlands | 0 | 0 | 0 | 1 | 0 | 1 |
|  | New Zealand | 0 | 0 | 2 | 0 | 3 | 5 |
|  | Norway | 0 | 0 | 0 | 2 | 0 | 2 |
|  | Philippines | 0 | 0 | 1 | 0 | 0 | 1 |
|  | Portugal | 0 | 0 | 0 | 1 | 1 | 2 |
|  | Romania | 0 | 0 | 1 | 0 | 0 | 1 |
|  | Singapore | 0 | 0 | 0 | 1 | 0 | 1 |
|  | South Africa | 0 | 0 | 0 | 1 | 0 | 1 |
|  | Spain | 0 | 0 | 1 | 0 | 1 | 2 |
|  | Switzerland | 0 | 0 | 0 | 0 | 2 | 2 |
|  | United Kingdom of Great Britain and Northern Ireland | 19 | 54 | 58 | 43 | 71 | 245 |
|  | United States of America | 12 | 8 | 20 | 37 | 26 | 103 |
| Total | | 32 | 73 | 100 | 119 | 121 | 445 |

*Supplementary Table 3: Satisfaction with mental health services by country.*
